# TWIST interacts with endothelin-1/endothelin A receptor signaling in osteosarcoma cell survival against cisplatin

**DOI:** 10.3892/ol.2013.1111

**Published:** 2013-01-07

**Authors:** YONG ZHOU, XIAOFANG ZANG, ZUFA HUANG, CHAOYUE ZHANG

**Affiliations:** Department of Orthopaedics, The Third Xiangya Hospital, Central South University, Hunan, Changsha 410013, P.R. China

**Keywords:** TWIST, endothelin-1, endothelin A receptor, osteosarcoma, cell survival, cisplatin

## Abstract

Both TWIST and the endothelin-1 (ET-1)/endothelin A receptor (ETAR) signaling are important in osteosarcoma (OS) progression. In the present study, the interaction between TWIST and ET-1/ETAR signaling in OS cells was investigated, and the impact of the functional interaction on OS cell survival against chemotherapy agent-induced apoptosis was assessed. TWIST was overexpressed and knocked down in Saos-2 and MG-63 OS cells, respectively. In Saos-2 cells, overexpression of TWIST significantly decreased ET-1 mRNA and protein expression levels, cell survival against cisplatin and phosphorylation of Akt at serine 473 (ser473), which was abolished by the selective phosphatidylinositol 3-kinase (PI3K) inhibitor, LY294002, or the selective ETAR inhibitor, BQ123. In MG-63 cells, knockdown of TWIST significantly increased ET-1 expression, cell survival against cisplatin and phosphorylation of Akt at ser473. However, exogenous ET-1 only partially rescued cell survival against cisplatin-induced apoptosis in the cells in which TWIST had been knocked down in the presence of LY294002. In conclusion, we have demonstrated that TWIST significantly, although only partially, decreases OS cell survival against cisplatin by downregulating ET-1/ETAR signaling via inhibition of the PI3K/Akt pathway. To the best of our knowledge, the present study has provided the first evidence of a functional interaction between TWIST and ET-1/ETAR signaling in OS cells. This finding adds novel insights into the molecular mechanisms underlying OS progression, cell survival and chemoresistance.

## Introduction

Osteosarcoma (OS) is the most common primary bone malignancy, comprising ∼35% of all types of bone cancer. This disease is the eighth most common type of cancer among children; it accounts for 2.4% of all pediatric malignancies (1). For patients with OS, the use of chemotherapy with surgical resection alone has improved survival from 11% in the 1960s, to 70% by the mid-1980s (2). However, survival has since plateaued, regardless of advances in anticancer therapy (2). Elucidation of the mechanisms of chemoresistance and implementation of strategies to overcome chemoresistance will likely be pivotal to improving survival in OS patients.

TWIST, also known as TWIST1, belongs to the basic helix-loop-helix (bHLH) transcription factor family. During embryonic development, TWIST is essential in the specification of the mesoderm and the differentiation of mesoderm-derived tissues (3). TWIST haploinsufficiency has been demonstrated to impair bone development in both mice and humans (4,5). A high expression of TWIST has been detected in several types of cancer and has been associated with the initial phase of metastatic progression (6). By contrast, in a homogeneous cohort of OS patients (7), the *TWIST* gene was frequently deleted in the tumors at diagnosis, and its haploinsufficiency was significantly correlated with a poorer patient outcome (3).

Endothelin-1 (ET-1), which promotes tumor cell proliferation and survival through the endothelin A receptor (ETAR), is expressed in a range of malignancies (8). Both ET-1 and ETAR are expressed in OS cells and tissue (9,10). Felx *et al* demonstrated that ET-1 is important in OS metastasis; it may promote OS cell invasion by inducing the synthesis of MMP-2 through ETAR (9). Zhao *et al* revealed that increased ET-1 expression was associated with an increased malignancy of OS, and that ET-1 promoted OS cell invasion and survival against cisplatin-induced apoptosis through ETAR, suggesting that ET-1/ETAR signaling may be a potential therapeutic target for OS metastasis as well as a target for overcoming OS cell chemoresistance (9,10).

We have performed the first study that investigates the interaction between TWIST and ET-1/ETAR signaling in OS cells, and we have analyzed how this functional interaction may affect OS cell survival against chemotherapy agent-induced apoptosis.

## Materials and methods

### Cells lines, plasmids and reagents

The human OS cell lines, Saos-2 and MG-63, were purchased from the American Type Culture Collection (Rockville, MD, USA). Human TWIST cDNA was subcloned into a pcDNA 3.1 expression vector as described previously by Matsuo *et al*(11). The following were purchased from Santa Cruz Biotechnology, Inc. (Santa Cruz, CA, USA): TWIST (sc-38604-V) short hairpin RNA (shRNA) lentiviral particles, control shRNA lentiviral particles-A (sc-108080), anti-TWIST (sc-81417) antibody, anti-ET-1 (sc-21625) antibody, anti-Akt (ser473) (sc-24500) antibody and anti-P-Akt (ser473) (sc-101629) antibody. All secondary antibodies were purchased from Jackson ImmunoResearch Laboratories, Inc. (West Grove, PA, USA). The ET-1 enzyme-linked immunosorbent assay (ELISA) kit was purchased from R&D Systems (Minneapolis, MN, USA), while the DeadEnd™ Fluorometric terminal deoxynucleotidyl transferase mediated nick-end labeling (TUNEL) system was purchased from Promega (Madison, WI, USA) and the Superfect™ transfection reagent was purchased from Qiagen (Valencia, CA, USA). Puromycin, cisplatin, synthetic ET-1, LY294002, BQ123 and all chemicals of reagent grade were purchased from Sigma (St. Louis, MO, USA).

The study was approved by the ethics committee of Xiangya Hospital, Central South University, Changsha, China.

### Transfection and lentiviral transduction

The Superfect transfection reagent (Qiagen) was used to transfect Saos-2 cells according to the manufacturer’s instructions. Selection with puromycin (5 *μ*g/ml) was then employed to generate pools of stable transductants according to the manufacturer’s instructions. The *TWIST*-shRNA lentiviral particles contained expression constructs that encoded target-specific 19–25 nt (plus hairpin) shRNA designed to specifically knockdown *TWIST* gene expression; while the control shRNA lentiviral particles contained a scrambled shRNA sequence that was not able to inititate the degradation of any cellular mRNA, and was used as a negative control for *TWIST* shRNA lentiviral particles. Subsequently, Saos-2 and MG-63 cells were transduced with the lentiviral particles. Selection with puromycin (5 *μ*g/ml) was then used to generate pools of stable transductants according to the manufacturer’s instructions (Santa Cruz Biotechnology, Inc.).

### Real-time quantitative reverse transcription PCR

TRIzol reagent followed by purification with the TURBO DNA-free system (Ambion; Austin, TX, USA) was used to prepare RNA from brain tissue samples. The cDNA was synthesized using the SuperScript II reverse transcriptase (Invitrogen Life Technologies, Inc.; Carlsbad, CA, USA). Using the SYBR-Green I kit (Roche) as described by the manufacturer, real-time quantitative PCR was performed in the LightCycler thermal cycler system (Roche Diagnostics; Indianapolis, IN, USA). The results were normalized against those of the housekeeping gene glyceraldehyde-3-phosphate dehydrogenase (*GAPDH*) in the same sample. The following primer sequences were used: Forward: 5′-TCCTCTGCTGGTTCCTGACT-3′ and reverse: 5′-CAGAAACTCCACCCCTGTGT-3′ for human *ET-1*; forward: 5′-GACTCATGACCACAGTCCATGC-3′ and reverse: 5′-AGAGGCAGGGATGATGTTCTG-3′ for human *GAPDH*. Each experiment was repeated twice and performed in triplicate.

### Western blot analysis

The ET-1 ELISA kit was used to assess the secreted levels of ET-1 in the cell culture supernatants. In brief, cells were grown to confluence in 10-cm dishes in Roswell Park Memorial Institute (RPMI)-1640 medium supplemented with 10% fetal bovine serum (FBS), which was then replaced with serum-free medium. The cells were incubated for a further 16 h, and then cell culture supernatants were collected for ELISA according to the manufacturer’s instructions (R&D Systems). ELISA-detected ET-1 concentrations are presented as the fold change relative to that of the normal control cells (designated as 1) and were normalized against cell number (per 10^6^ cells). Each ELISA experiment was repeated three times and performed in duplicate. In the western blot analyses, protein was extracted using a lysis buffer comprising 150 mM NaCl; 2% Triton; 0.1% sodium dodecyl sulfate (SDS); 50 mM Tris, pH 8.0 and 10% protease inhibitor cocktail (Sigma), and stored at -20°C. Equal amounts of protein (25 *μ*g) for each sample were loaded onto pre-cast 7.5% Mini Protean TGX gels (Bio-Rad, Hercules, CA, USA). Prior to loading onto the gels, the proteins were quantified and equal loading was verified by Ponceau coloration. The proteins were separated by electrophoresis for 50 min at 200 V, then transferred onto a polyvinylidene fluoride (PVDF) transfer membrane (Amersham Biosciences/GE Healthcare; Piscataway, NJ, USA) for 55 min at 100 V. Membranes were incubated with a 1/500 dilution of anti-TWIST, anti-MMP-2 or anti-ET-1 antibody for 1 h. Following washing, secondary antibodies with horseradish peroxidase conjugate (1/5000; 1 h) were used to reveal the membranes. A GE Healthcare enhanced chemiluminescence (ECL) kit was used to reveal the peroxidase activity.

### Measurement of apoptosis by TUNEL assay

The TUNEL assay was performed using the DeadEnd Fluorometric TUNEL system according to the instructions provided by Promega. Cells were treated with cisplatin (10 nM) in the presence or absence of ET-1 (10 or 100 pM) and/or LY294002 (50 *μ*M) or BQ123 (5 *μ*M) for ≤8 h. A standard fluorescein filter was used to detect the green nuclear fluorescence emitted by apoptotic cells. A blue nuclear fluorescence was emitted by all cells stained with 4′,6-diamidino-2-phenylindole (DAPI). The relative number of apoptotic cells was determined by counting the number of TUNEL-positive cells in five random fields in each sample, by fluorescence microscopy of the slides (magnification, ×100).

### Statistical analyses

Statistical analyses were performed using the Statistical Package for the Social Sciences (SPSS)for Windows, version 10.0 (SPSS, Inc.; Chicago, IL, USA). Data values were expressed as mean ± standard deviation. A one-way analysis of variance (ANOVA) followed by post hoc pairwise comparisons using the least significant difference method were used to compare means between multiple groups. Two-tailed α=0.05 was used as the significance level for this study.

## Results

**Figure 1:**
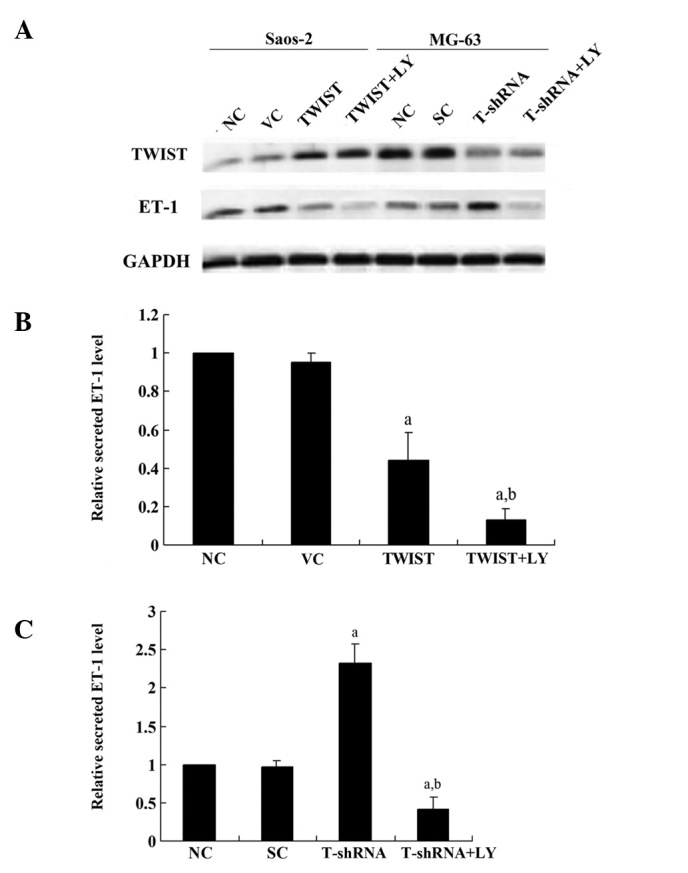
Western blot analysis of TWIST and endothelin-1 (ET-1) expression in Saos-2 and MG-63 osteosarcoma cells (A). The expression of TWIST in normal control cells (NC), cells stably transfected with empty pcDNA3 vector (VC) and cells stably transfected with pcDNA3-TWIST expression vector (TWIST) with or without LY294002 (LY; 50 mM) treatment was analyzed with western blot analysis in Saos-2 cells. Additionally, the expression of TWIST in NC, cells stably transduced with scrambled control short hairpin RNA (shRNA) (SC) and cells stably transduced with TWIST-shRNA (T-shRNA) with or without LY (50 *μ*M) treatment was analyzed with western blot analysis in MG-63 cells. The loading control used was GAPDH blotting. The secreted ET-1 level in cell culture supernatants in all the above experimental groups in Saos-2 cells (B) and MG-63 cells (C) was quantified using an enzyme-linked immunosorbant assay (ELISA) and was normalized against cell number (per 10^6^ cells). The secreted ET-1 level is presented as the fold change relative to that of the NC (designated as 1). ^a^P<0.05 compared with NC and VC (B) or SC (C); ^b^P<0.05 compared with TWIST (B) or T-shRNA (C).

TWIST is expressed at a very low level in Saos-2 cells (12), but is widely detectable in MG-63 cells (13). Saos-2 cells were stably transfected with a TWIST expression vector to overex-press TWIST, and MG-63 cells were stably transduced with TWIST-shRNA to knock down TWIST expression, in order to study the interaction between TWIST and ET-1 in OS cells. Fig. 1 demonstrates that TWIST was overexpressed >3-fold in the Saos-2 cells, and the endogenous level of TWIST was decreased by >75% in MG-63 cells, compared with the control cells. Additionally, ET-1 exhibited a higher constitutive level in Saos-2 cells than in MG-63 cells. Overexpression of TWIST decreased the ET-1 level ∼2-fold in Saos-2 cells compared with the controls, which was significantly strengthened by the selective phosphatidylinositol 3-kinase (PI3K) inhibitor LY294002. Knocking down TWIST increased the ET-1 level >2-fold in MG-63 cellscompared with the controls, which was abolished by LY294002. Similar secreted ET-1 level results were demonstrated in both cell lines, which suggests that TWIST inhibits ET-1 expression in a PI3K-dependent manner in OS cells.

**Figure 2:**
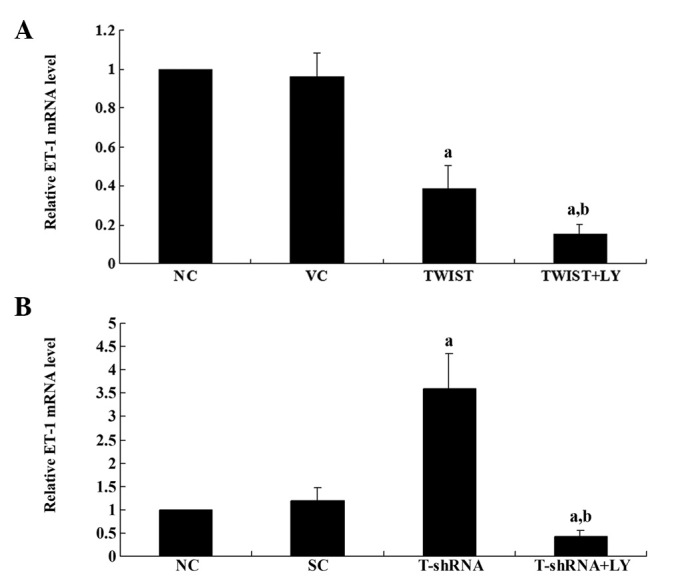
The level of endothelin-1 (ET-1) mRNA in Saos-2 and MG-63 cells (A). The ET-1 mRNA level in normal control cells (NC), cells stably transfected with empty pcDNA3 vector (VC) and cells stably transfected with pcDNA3-TWIST expression vector (TWIST) with or without LY294002 (LY; 50 mM) treatment was analyzed with real-time reverse transcription (RT)-PCR in Saos-2 cells. (B) The ET-1 mRNA level in NC, cells stably transduced with scrambled control short hairpin RNA (shRNA) (SC) and cells stably transduced with TWIST-shRNA (T-shRNA) with or without LY (50 *μ*M) treatment was analyzed with real-time RT-PCR in MG-63 cells. The ET-1 mRNA level is presented as the fold change relative to that of the NC (designated as 1). ^a^P<0.05 compared with NC and VC (A) or SC (B); ^b^P<0.05 compared with TWIST (A) or T-shRNA (B).

Real-time RT-PCR results showed that in Saos-2 cells, overexpression of TWIST decreased the ET-1 mRNA level >2-fold compared with the controls, which was strengthened by LY294002 (Fig. 2A). Additionally, in MG-6 cells, knockdown of TWIST increased the ET-1 mRNA level >3-fold compared with the controls, which was abolished by LY294002 (Fig. 2B). Therefore, TWIST inhibits the expression of ET-1 at the transcriptional level in a PI3K-dependent manner in OS cells.

**Figure 3:**
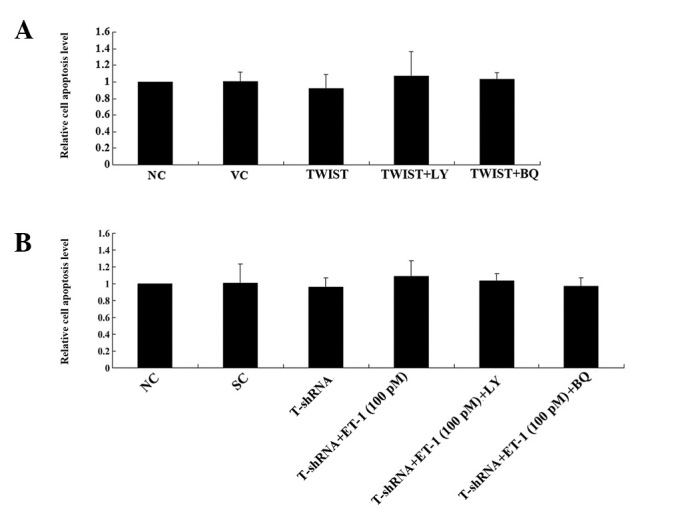
Cell apoptosis under normal culture conditions in Saos-2 and MG-63 cells. Saos-2 (A) and MG-63 (B) cells were placed under normal culture conditions for 8 h. The percentage of terminal deoxynucleotidyl transferase mediated nick-end labeling (TUNEL)-positive cells in total cells after 8 h was used to determine the cell apoptosis rate. TUNEL assays were performed in normal control cells (NC), cells stably transfected with empty pcDNA3 vector (VC) and cells stably transfected with a pcDNA3-TWIST expression vector (TWIST) with or without LY294002 (LY; 5 *μ*M) treatment in Saos-2 cells. TUNEL assays in NC, cells stably transduced with scrambled control short hairpin RNA (shRNA) (SC) and cells stably transduced with TWIST-shRNA (T-shRNA) with or without LY (50 *μ*M) treatment in MG-63 cells. The cell apoptosis level is presented as the fold change relative to that of the NC (designated as 1).

**Figure 4:**
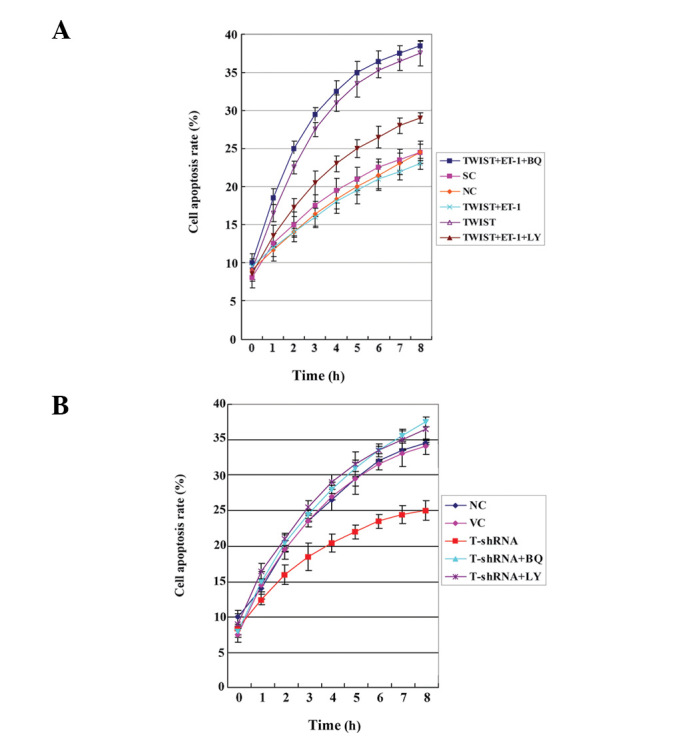
Cell apoptosis in Saos-2 (A) and MG-63 (B) cells treated with 10 nM cisplatin for 8 h. Terminal deoxynucleotidyl transferase mediated nick-end labeling (TUNEL) assays were performed in normal control cells (NC), cells stably transfected with empty pcDNA3 vector (VC), cells stably transfected with pcDNA3-TWIST expression vector (TWIST) with or without LY294002 (LY; 50 *μ*M) or BQ123 (BQ; 5 *μ*M) in Saos-2 cells. TUNEL assays were performed in normal control cells (NC), cells stably transduced with scrambled control short hairpin RNA (shRNA) (SC), and cells stably transduced with TWIST-shRNA (T-shRNA) with or without ET-1 (100 pM) treatment alone or ET-1 (100 pM) combined with LY (50 *μ*M) or BQ (5 *μ*M) in MG-63 cells. The cell apoptosis rate is presented as the percentage of TUNEL-positive cells in total cells.

Subsequently, to investigate the effect of the interaction between TWIST and ET-1/ETAR signaling on OS survival, the cell apoptosis rate in both cell lines treated with 10 nM of cisplatin was examined. Cisplatin is an apoptosis-inducing chemotherapeutic agent typically used in OS treatment. Under normal culture conditions, overexpressing or knocking down TWIST in the presence or absence of ET-1 (100 pM) and/or LY294002 (50 *μ*M) or BQ123 (5 *μ*M) for ≤8 h was not observed to signifcantly affect the rate of cell apoptosis (Fig. 3). However, in Saos-2 cells treated with cisplatin, overexpressing TWIST led to a significantly increased cell apoptosis rate compared with the controls, which was reversed by exogenous ET-1 (100 pM). Additionally, the selective ETAR inhibitor (BQ123) was able to completely block the rescue effect of ET-1, while LY294002 partially blocked the rescue effect (Fig. 4A). In MG-63 cells treated with cisplatin, knocking down TWIST significantly decreased the cell apoptosis rate. This effect was was abolished by BQ123 or LY294002 (Fig. 4B).

**Figure 5:**
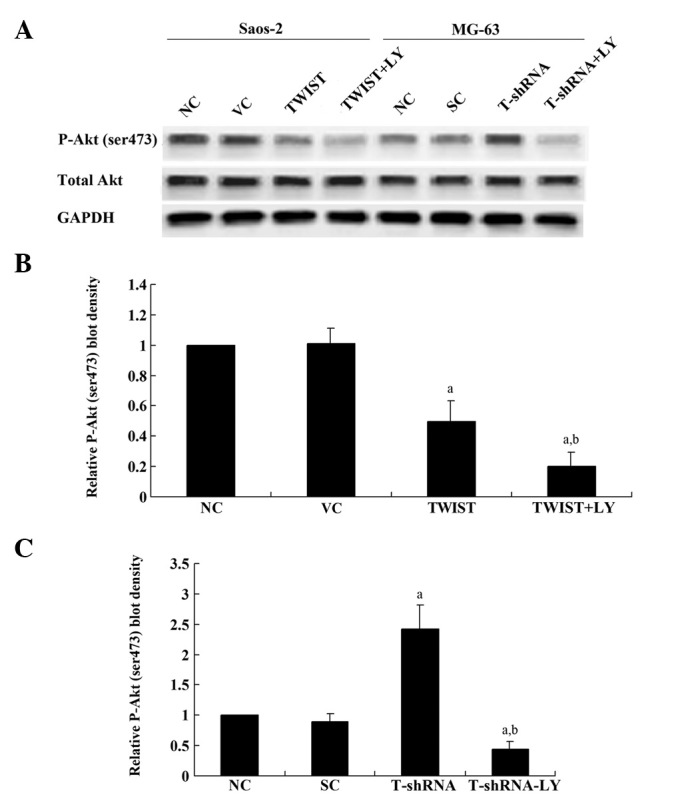
Western blot analysis of the phosphorylated Akt (P-Akt) level in Saos-2 and MG-63 cells. (A) The level of total Akt and P-Akt at serine 473 (ser473) in normal control cells (NC), cells stably transfected with empty pcDNA3 vector (VC), and cells stably transfected with pcDNA3-TWIST expression vector (TWIST) with or without LY294002 (LY; 50 *μ*M) treatment was analyzed with western blot analysis in Saos-2 cells. (B) The level of total Akt and P-Akt at ser473 in NC, cells stably transduced with scrambled control short hairpin RNA (shRNA) (SC) and cells stably transduced with TWIST-shRNA (T-shRNA) with or without LY (50 *μ*M) treatment was analyzed with western blot analysis in MG-63 cells. GAPDH blotting was used as a loading control. P-Akt (ser473), total Akt and GAPDH blots were measured by densitometry. The density of the P-Akt (ser473) blot was normalized against that of total Akt and GAPDH to obtain a relative P-Akt (ser473) blot density, which was expressed as the fold change compared with the relative P-Akt (ser473) blot density of NC (designated as 1). ^a^P<0.05 compared with NC and VC (B) or SC (C); ^b^P<0.05 compared with TWIST (B) or T-shRNA (C).

The results suggest that TWIST significantly decreases OS cell survival against cisplatin by inhibiting ET-1/ETAR signaling, which functions downstream of PI3K. The PI3K/Akt pathway has been demonstrated to activate *ET-1* gene transcription (14), in accordance with our finding that the regulatory effects of TWIST on ET-1 expression and ET-1/ETAR signaling may be completely blocked by LY294002. Thus, we subsequently tested the effect of overexpressing or knocking down TWIST on the PI3K/Akt survival signaling pathway. In Saos-2 cells, overexpression of TWIST significantly decreased phosphorylation at serine 473 (ser473) of Akt, which is required for complete activation of Akt; LY294002 treatment further decreased phosphorylation at this site (Fig. 5A and B). In MG-63 cells, knockdown of TWIST increased the phosphorylation of Akt (P-Akt) at ser473 ≥2-fold compared with the controls, which was abolished by LY294003 (Fig. 5A and C). Overall, our results suggest that TWIST downregulates ET-1/ETAR signaling by inhibiting the PI3K/Akt pathway.

## Discussion

The ET-1/ETAR signaling pathway is considered to be a potential therapeutic target in the control of OS metastasis and chemoresistance (9,10). Although a high expression of TWIST has been described in several types of cancer and is associated with the initial phase of metastatic progression, the *TWIST* gene is frequently deleted in OS at diagnosis, and its haploinsufficiency is significantly correlated with a poorer patient outcome (6). In the present study, to our knowledge, we have conducted the first investigation into the functional interaction between TWIST and ET-1/ETAR signaling in OS cells. We assessed the effect of this interaction on OS cell survival against chemotherapy agent-induced apoptosis.

Saos-2 cells express a low level of TWIST and have been used as a model to investigate TWIST function (12). By contrast, TWIST is amply expressed in MG-63 cells (13). Therefore, overexpression and knockdown of TWIST were performed in the two cell lines, respectively, to approach the study objectives. The results of our study demonstrated that Saos-2 cells expressed a relatively higher constitutive level of ET-1 compared with MG-63 cells, suggesting that the constitutive levels of TWIST and ET-1 in OS cells may be negatively correlated. Further studies with additional OS cell lines are required to address the issue. In accordance with these findings, knockdown of TWIST in MG-63 cells increased the ET-1 expression level as well as cell survival against cisplatin, which was reversed by a PI3K inhibitor. Our results were concordant with those of previous studies; they demonstrated that TWIST regulates ET-1 expression at the transcriptional level in a PI3K-dependent manner in OS cells. Guenou *et al* demonstrated that TWIST haploinsufficiency results in PI3K accumulation and activation of PI3K/Akt-dependent osteoblast growth (14), while Kim *et al* revealed that activated PI3K was required for the activation of AP-1 and subsequent ET-1 gene transcription (15). In the present study, to our knowledge, we have provided the first evidence that TWIST downregulates ET-1 expression by inhibiting the PI3K/Akt pathway in OS cells.

Both TWIST and ET-1/ETAR signaling reportedly promote tumor cell survival and chemoresistance in human cancer (16–18). However, our results suggest that TWIST decreases cell survival against chemotherapy agents by down-regulating ET-1/ETAR signaling, which is in accordance with the study demonstrating that *TWIST* haploinsufficiency was significantly correlated with a poorer patient outcome (3). ET-1 itself is an activator of the PI3K/Akt pathway (19,20), while TWIST inhibits it, which explains why exogenous ET-1 was able to completely restore cell survival against cisplatin in OS cells overexpressing TWIST. However, exogenous ET-1 was only partially able to rescue cell survival in OS cells overexpressing TWIST in the presence of an extremely high concentration of LY294002, implicating the involvement of other signaling pathways downstream of PI3K/Akt (i.e., other than ET-1/ETAR signaling) in the inhibitory effects of TWIST on OS cell survival against chemotherapy agents.

Cisplatin elicits DNA repair mechanisms by cross-linking DNA, and this activates apoptosis when repair is not possible (21). It is not yet understood whether the functional interaction between TWIST and ET-1 is able to affect OS cell survival against additional types of chemotherapy agents. Further studies with further types of chemotherapy agents and OS cell lines would elaborate this issue.

In conclusion, we demonstrate that TWIST significantly decreases OS cell survival against cisplatin by downregulating ET-1/ETAR signaling via inhibition of the PI3K/Akt pathway. To our knowledge, our study provides the first evidence of a functional interaction between TWIST and ET-1/ETAR signaling in OS cells, providing novel insights into the molecular mechanisms that underlie OS progression, cell survival and chemoresistance.
